# Disgust-specific impairment of facial emotion recognition in Parkinson’s disease patients with mild cognitive impairment

**DOI:** 10.1093/scan/nsae073

**Published:** 2024-10-17

**Authors:** Ke-Wei Chiang, Chun-Hsiang Tan, Wei-Pin Hong, Rwei-Ling Yu

**Affiliations:** Institute of Behavioral Medicine, College of Medicine, National Cheng Kung University, Tainan 701, Taiwan; Department of Psychiatry, China Medical University Hospital, China Medical University, Taichung 404327, Taiwan; Department of Neurology, Kaohsiung Medical University Hospital, Kaohsiung Medical University, Kaohsiung 807378, Taiwan; Graduate Institute of Clinical Medicine, College of Medicine, Kaohsiung Medical University, Kaohsiung 807378, Taiwan; Department of Neurology, National Cheng Kung University Hospital, College of Medicine, National Cheng Kung University, Tainan 701401, Taiwan; Institute of Behavioral Medicine, College of Medicine, National Cheng Kung University, Tainan 701, Taiwan; Office of Strategic Planning, National Cheng Kung University, Tainan 701401, Taiwan

**Keywords:** cognitive impairment, cognitive function, disgust facial emotion, emotion recognition, Parkinson’s disease

## Abstract

This study investigated the association between cognitive function and facial emotion recognition (FER) in patients with Parkinson’s disease (PD) and mild cognitive impairment (PD-MCI). We enrolled 126 participants from Taiwan, including 63 patients with idiopathic PD and 63 matched healthy controls. The PD group was divided into two groups: those with normal cognitive function (PD-NC) and those with MCI (PD-MCI). Participants underwent a modality emotion recognition test and comprehensive cognitive assessment. Our findings reveal that patients with PD-MCI exhibit significantly impaired FER, especially in recognizing “disgust,” compared with patients with PD-NC and healthy adults (*P* = .001). This deficit correlates with executive function, attention, memory, and visuospatial abilities. Attention mediates the relationship between executive function and “disgust” FER. The findings highlight how patients with PD-MCI are specifically challenged when recognizing “disgust” and suggest that cognitive training focusing on cognitive flexibility and attention may improve their FER abilities. This study contributes to our understanding of the nuanced relationship between cognitive dysfunction and FER in patients with PD-MCI, emphasizing the need for targeted interventions.

## Introduction

### Impaired facial emotion recognition in Parkinson’s disease

Facial emotion recognition (FER) is crucial for understanding the emotional states of others and establishing interpersonal connections. It involves the perception of emotions, which is mediated by specific components of the ventral system in the brain (Moonen et al. 2017). Humans perform differently at distinguishing different facial emotion categories. For instance, Caruana et al. (2020) presented that people are more likely to gain a conscious connection with the threatening facial emotion of “fear,” and if the stimulus were presented gaze-directly to the participants, they would have better performance in distinguishing emotions.

FER relies on well-defined neurocognitive systems, including the amygdala, right anterior temporal pole, insula, and right posterior fusiform gyrus (Hsieh et al. 2012). Pitcher and Ungerleider presented evidence for a visual pathway on the lateral brain surface that is engaged in the dynamic aspects of social perception (including FER and eye gaze discrimination), which projects from the early visual cortex via motion-selective areas into the superior temporal sulcus (STS; Pitcher and Ungerleider 2021). Vaessen et al. presented a multi-stimulus and multimodal perception of emotion to determine the neuropathology of emotion recognition. They found that the superior temporal sulcus, cingulate gyrus, and angular gyrus could more accurately distinguish the visual modality of emotion (including the face and body) (Vaessen et al. 2023).

Different patterns of neurodegenerative diseases are associated with different neuropathologies of emotion recognition. The inability to recognize negative emotions in patients with Alzheimer’s disease may be due to emotional memory defects caused by hippocampal dysfunction, and these defects appear in the early stages of the disease (Chaudhary et al. 2022). Emotion recognition deficits in patients with frontotemporal lobar degeneration (FTLD) are associated with reduced gray matter (GM) volume in the amygdala, insula, inferior frontal lobe, medial prefrontal lobe, and orbitofrontal cortex (Downey et al. 2015). Furthermore, based on functional magnetic resonance imaging, patients with FTLD have been shown to have less fusiform gyrus activation when watching videos of emotional facial expressions (Marshall et al. 2019).

Owing to the neurocognitive impairments associated with Parkinson’s disease (PD), individuals affected by this chronic and progressive neurodegenerative disorder may face challenges in perceiving and interpreting others’ emotions (Armstrong and Okun 2020). PD is characterized by the deterioration of dopaminergic neurons in the substantia nigra pars compacta, resulting in motor symptoms, such as tremors, rigidity, and bradykinesia, and non-motor symptoms (Lin et al. 2019, Yu et al. 2021, Chang et al. 2024, Yi et al. 2024).

Studies have provided evidence of impaired FER ability in patients with PD, particularly for neutral or negative emotions (Argaud et al. 2018, Yu et al. 2018). Such deficits may affect the patients’ social functioning (Su et al. 2020, Chuang et al. 2021). The mesolimbic dopamine pathway is a possible brain mechanism affecting FER in patients with PD (Péron et al. 2012). The ability to distinguish specific emotions such as “sadness,” “anger,” and “disgust” in patients with PD is related to the activity of certain brain regions. For instance, the right orbitofrontal cortex, amygdala, and posterior central sulcus aid in identifying “sad” emotions. The recognition of “anger” emotions involves the right fusiform gyrus, ventral striatum, and subcortical regions, whereas the dorsal cingulate cortex and striatum are responsible for identifying “disgust” emotions (Ibarretxe‐Bilbao et al. 2009). Suzuki et al. (2006) suggested that PD selectively impairs FER performance for specific emotions, particularly “disgust” (Suzuki et al. 2006). Furthermore, Motomura et al. (2019) documented that aberrant insula reactions, particularly within the anterior to the middle insula, exhibit a correlation with both FER of “disgust” and cognitive deterioration (Motomura et al. 2019). These outcomes suggest that discerning “disgust” may necessitate a greater degree of cognitive intervention than discerning other emotional states.

### Factors influencing FER in PD

The FER of patients with PD may be influenced by various factors such as age (Yu et al. 2018), sex (Yu et al. 2018), motor symptoms (Chuang et al. 2021), and cognitive function (Alonso-Recio et al. 2014, Siquier and Andrés 2022). Alonso-Recio et al. (2014) and Siquier and Andrés (2022) suggested that inhibition dysfunction in patients with PD significantly affects their FER performance and that cognitive function plays a crucial role in this ability. Attention has also been found to impact FER performance, particularly selective attention (Alonso-Recio et al. 2014, Siquier and Andrés 2022). Patients may commonly exhibit a spectrum of symptoms, such as cognitive dysfunction (Fang et al. 2019, Chen et al. 2021), social brain dysfunction, neuropsychiatric symptoms (Chen et al. 2022), and sleep disturbance (Yu et al. 2015). Ho et al. (2020) found that patients with PD and full-blown dementia had significantly lower FER performance than healthy individuals, particularly in recognizing negative emotions.

### FER in PD with mild cognitive impairment

Mild cognitive impairment (MCI) is a precursor of dementia and is of significant interest in preventive medicine (Yu et al. 2020, 2022b, Yu and Wu 2022). The diagnostic criteria for MCI were established by Litvan et al. (2012), and the prevalence of patients with PD and MCI (PD-MCI) is ∼40% (Baiano et al. 2020). However, few studies have explored the relationship between FER and cognitive function in patients with PD-MCI. Pietschnig et al. (2016) found that patients with normal cognitive function had better FER performance than patients with nonamnestic-MCI and amnestic-MCI; they found that better FER performance was associated with better language and executive function (Pietschnig et al. 2016). Conversely, Herrera et al. (2011) suggested that FER may be linked to nonlanguage deficits (Herrera et al. 2011). Subsequently, Waldthaler et al. (2019) suggested that oculomotor dysfunction could affect FER performance in individuals with PD-MCI. This compromised visual exploration has been linked to impaired emotion recognition in PD, even without cognitive impairment (Waldthaler et al. 2019). Galtier et al. (2021) found that patients with PD-MCI and PD with subjective cognitive decline had lower overall FER scores than healthy controls, and patients with PD-MCI made misjudgments in recognizing faces, even when presented with frontal images (Galtier et al. 2021). Burgio et al. (2024) found that the performance of facial-matching tasks (including anger, fear, sad, surprise, and happy) in patients with PD-MCI correlated with their scores in attention, executive functions, and general cognitive function (Burgio et al. 2024).

### The impact of cognitive dysfunction on specific emotion categories in PD-MCI

Previous research has found that individuals with PD-MCI exhibit significantly poorer total FER performance than healthy controls. However, the specific impact of cognitive dysfunction on each emotion category of FER, including neutral, happy, sad, fear, disgust, anger, and surprise, in patients with PD-MCI remains unclear. Based on the brain mechanism (Hsieh et al. 2012, Moonen et al. 2017), we hypothesized that the FER ability of patients with PD-MCI is different and that each emotion is related to different cognitive performances. Therefore, we aimed to explore FER performance in patients with PD-MCI and the role of cognitive function in FER.

## Methods

### Participants

A total of 126 participants were recruited from February 2019 to May 2021 in southern Taiwan, including 63 patients with idiopathic PD (aged 47–78 years, mean = 62.97 years) and 63 age-, sex-, and education-matched healthy controls (aged 50–79 years, mean = 63.14 years). A neurologist diagnosed patients with PD according to the clinical diagnostic criteria established by the UK PD Society Brain Bank. The PD group was selected based on the inclusion criterion of patients diagnosed with idiopathic PD. The exclusion criteria were as follows: atypical Parkinsonism, impaired reading ability, illiteracy, color blindness, history of stroke or brain trauma, hepatitis, undergoing chemotherapy, and any other factors that may hinder the completion of the test. The control group comprised healthy individuals [healthy control (HC) group] recruited from various community settings. The inclusion criterion for the HC group was a Mini-Mental State Examination score of >24, and the exclusion criteria were the same as those for the PD group. According to the level II diagnostic criteria for PD-MCI (Movement Disorder Society; MDS; Litvan et al. 2012), patients with PD were divided into two subgroups: the PD-MCI and patients with PD with normal cognitive function (PD-NC) groups. All participants provided written informed consent before the study commenced, and all experiments were conducted following the Declaration of Helsinki of 1975. The ethical research committees of the hospitals [including National Cheng Kung University Hospital (A-ER-107-425) and Kaohsiung Medical University Hospital (KMUHIRB-G(II)-20160001)] approved the study protocols. We confirm that we have read the journal’s guidelines on issues involved in ethical publication and affirm that this work is consistent with these guidelines.

### Measurement

All the participants were interviewed to record their demographics, general cognitive function, and clinical characteristics. General cognitive function was measured using the Montreal Cognitive Assessment (Nasreddine et al. 2005). The Hoehn–Yahr stage and MDS-Unified PD Rating Scale were used to evaluate the severity of PD and motor symptoms (Yu et al. 2017). According to the level II of the PD-MCI diagnostic criteria recommendation from MDS (Litvan et al. 2012), the neuropsychological tests (Lezak et al. 2004) were applied to assess the five cognitive domains, each containing at least two different tests (Table 1).

**Table 1.** Neuropsychological tests and the cognitive domain to which each neuropsychological test belongs

Attention/working memory	Digit Span (DS) of Wechsler Intelligence Scale-Third edition (WAIS-III) Stroop Task-Word score (Stroop-W) and Stroop Task-Color score (Stroop-C)
Executive function	Color Trails Test-part B (CTT-B) category(C), perseverative error (PE), and non-perseverative error (NPE) of the Modified Card Sorting Test (MCST)
Memory function	immediate (LM-I) and delayed (LM-II) recall of the logical memory test of the Wechsler Memory Scales-Third edition (WMS-III) immediate (VR-I) and delayed (VR-II) recall of the visual reproduction test of the WMS-III
Visual-spatial function	Judgment of Line Orientation (JLO) Mini-Mental State Examination (MMSE)-pentagons
Language function	Verbal fluency (VF) MMSE-Language

We used the FER subtests (i.e. Subtest 3 and Subtest 4) of the MMER app (Yu et al. 2022a) as the measurement of FER, with one point for each question. The two subtests were identification tests; this FER test mode is the most commonly used (Argaud et al. 2018). In Subtest 3, participants were presented with a face on a tablet along with seven emotional words: neutral, happy, sad, anger, disgust, fear, and surprise. They were required to select the word that best matched the facial expressions. This subtest included 30 questions, broken down as follows: four neutral, three happy, three sad, five angry, six disgusted, six fearful, and three surprised. In Subtest 4, the setup was reversed, displaying an emotional word with seven corresponding faces. Participants were required to identify a face that accurately represented a given emotion. This subtest consisted of 20 questions distributed as follows: two questions each for neutral and happy, three questions each for sad and angry, four questions each for disgust and fear, and two questions for surprise. Each correct response in these subtests earned the participants one point.

### Statistical analysis

The confidence interval was set to 95%, which contained an error of 5%, and statistical significance was set at *P* < .05. Bonferroni correction was applied to adjust the *P*-value for multiple comparisons.

The Kolmogorov–Smirnov test was used to examine data normality. Comparisons between groups were performed using the Mann–Whitney *U*-test and Kruskal–Wallis test when the data were non-normally distributed, and the independent sample *t*-test was used when the data were normally distributed. The chi-squared test was used when variables were categorical. The post-hoc was performed using Scheffe’s method.

A regression model was established to investigate whether the cognitive impairment indices of the patients could predict their FER ability within each emotion category. Demographic variables such as age, sex, and education, as well as variables previously shown to be associated with deficits in FER in patients with PD (Yu et al. 2018), were included as covariates in the analysis. The number of deficits in the individual cognitive function index was used as the independent variable, and the performance of each category of emotion recognition ability was used as the dependent variable. Hierarchical linear regression was used to obtain the explanatory power of the indices of cognitive impairment on FER ability in each category.

Mediated regression analyses explored the relationships between executive function, other cognitive functions, and FER performance. Executive function indicators were used as independent variables, other cognitive indices were used as mediating variables, and FER performance was used as the dependent variable to obtain the mediation model.

### Transparency and openness

We report how we determined our sample size, all data exclusions (if any), all manipulations, and all measures in the study, following the Journal Article Reporting Standards (Kazak 2018). Data were analyzed using the 26th edition of Statistical Product and Service Solutions statistical software. This study’s Materials and Analysis Code is unavailable owing to ethical problems. The study design and analyses have not been registered previously.

## Results

**Figure 1.**
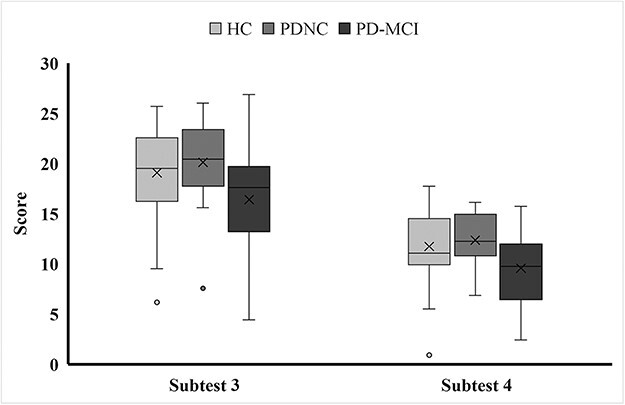
The performance of FER ability between study groups.

**Figure 2.**
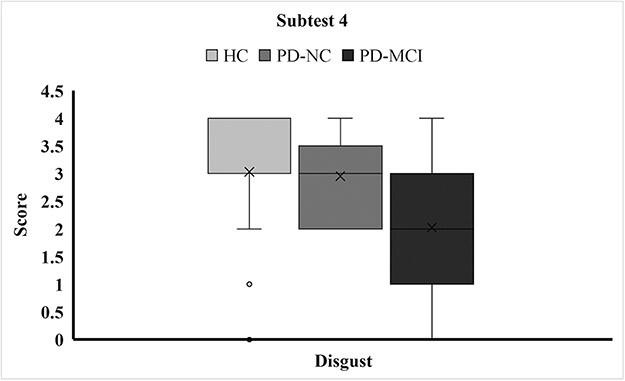
The performance of disgust FER ability between study groups.

The demographic and clinical characteristics of the participants are presented in Table 2. For the following reasons, we did not exclude 1–2 poor performances of HCs in Figs 1 and 2. First, according to the standard deviation method (Howell et al. 1998), these HC participants did not meet the criteria for exclusion based on this statistical definition. Second, our findings indicate that their inclusion did not introduce significant anomalies or distort the overall outcomes. Finally, the HC and PD participants were closely matched regarding key demographic and cognitive function variables. This matching is crucial for maintaining comparability and balance between the groups.

**Table 2.** Demographic and clinical characteristics in the study groups^+^

	HC (*n* = 63)	PD (*n* = 63)	Stat. value	*P-value*	Post-hoc	HC (*n* = 63)	PD-NC (*n* = 21)	PD-MCI (*n* = 42)	Stat. value	*P*-value*	Post-hoc
Male (%)	55.56	68.25	^a^2.154	.142	–	55.56	76.19	64.28	^a^2.995	.224	–
Education	13.11 (2.99)	12.96 (3.31)	^b^–0.177	.860	–	13.11 (2.99)	14.12 (2.99)	12.38 (3.34)	^c^4.728	.094	–
Age	63.14 (8.72)	62.97 (8.49)	^d^0.114	.910	–	63.14 (8.72)	60.95 (7.14)	63.98 (9.01)	^e^0.875	.419	–
MoCA	25.81 (2.89)	23.73 (3.13)	^b^–3.810	<.001	HC > PD	25.81 (2.89)	25.33 (1.88)	22.93 (3.34)	^c^21.493	<.001	HC = PD-NC > PD-MCI
Duration	–	6.14 (4.97)	–	–	–	–	5.88 (4.12)	6.27 (5.38)	^b^–0.190	.849	–
LEDD	–	535.15 (389.36)	–	–	–	–	412.98 (269.86)	596.24 (426.91)	^d^–.792	.078	–
MDS-UPDRS	–	44.51 (15.39)	–	–	–	–	42.95 (15.87)	45.29 (15.28)	^d^0.642	.521	–
PART I	–	4.57 (3.61)	–	–	–	–	5.00 (2.77)	4.36 (3.98)	^d^0.663	.510	–
PART II	–	6.37 (5.37)	–	–	–	–	6.00 (4.94)	6.55 (5.62)	^b^0.410	.682	–
PART III	–	32.57 (10.96)	–	–	–	–	30.95 (11.72)	33.38 (10.61)	^b^1.139	.255	–
Stage	–	1.81 (0.62)	–	–	–	–	1.81 (0.51)	1.81 (0.67)	^b^0.026	.980	–
**Attention/working memory**											
Stroop-W	–	76.71 (2.35)	–	–	–	–	86.05 (16.79)	72.05 (17.97)	^e^2.978	.004	–
Stroop-C	–	57.25 (2.01)	–	–	–	–	66.71 (14.72)	52.52 (14.44)	^e^3.652	<.001	PD-NC > PD-MCI
WAIS-DS	–	11.59 (0.33)	–	–	–	–	12.62 (2.82)	11.07 (2.40)	^c^−2.148	.032	
**Executive function**											
CTT-B	–	145.61 (6.56)	–	–	–	–	131.10 (24.76)	152.86 (60.34)	^c^0.744	.457	–
MCST-C	–	4.04 (0.22)	–	–	–	–	4.65 (0.87)	3.74 (1.98)	^c^−1.349	.177	–
MCST-PE	–	8.12 (1.25)	–	–	–	–	4.25 (2.63)	10.05 (11.60)	^c^2.034	.042	–
MCST-NPE	–	8.36 (0.56)	–	–	–	–	7.83 (2.88)	8.63 (5.10)	^e^4.461	.039	–
**Memory function**											
LM-I	–	10.56 (0.42)	–	–	–	–	11.81 (3.37)	9.93 (3.14)	^c^−1.929	.054	–
LM-II	–	10.78 (0.40)	–	–	–	–	12.24 (3.33)	10.05 (2.91)	^c^−2.225	.026	—
VR-I	–	10.79 (0.35)	–	–	–	–	11.62 (2.44)	10.38 (2.87)	^e^0.544	.464	–
VR-II	–	10.29 (0.39)	–	–	–	–	11.86 (3.26)	9.50 (2.79)	^c^−2.745	.006	–
**Visual-spatial function**											
JLO	–	22.09 (0.62)	–	–	–	–	25.31 (2.61)	20.48 (5.09)	^e^8.847	.004	–
pentagon^M^		0.83 (0.05)	–	–	–	–	1.00 (0.00)	0.74 (0.45)	^c^−2.561	.010	–
**Language function**											
VF	–	32.94 (1.05)	–	–	–	–	34.62 (5.32)	32.10 (9.43)	^e^3.658	.060	–
Language^M^	–	4.81 (0.05)	–	–	–	–	4.95 (0.22)	4.74 (0.5)	^c^−1.871	.061	–

Notes: ^+^Data are represented as mean and standard deviation;

M subtest of MMSE;

a Chi-square test;

b Mann–Whitney U-test;

c Kruskal–Wallis test;

d Independent sample *t*-test;

e One-way ANOVA;

HC = Healthy controls; PD = Parkinson’s disease; PD-NC = PD patients with normal cognition; PD-MCI = PD patients with Mild Cognitive Impairment; MoCA = Montreal Cognitive Assessment; LEDD = Levodopa Equivalent Daily Dose; MDS-UPDRS = Movement Disorder Society-sponsored revision of the Unified Parkinson’s Disease Rating Scale; H&Y stage = Hoehn-Yahr stage;

* Bonferroni correction was applied with a *P*-value < .0019.

### FER ability in PD patients

There was no significant difference in the total scores between the two groups (i.e. the PD and HC groups). However, we found a significant difference between the three study groups in the total score on Subtest 4 (*P *= .001; Fig. 1). The post hoc analysis revealed that the PD-NC and HC groups had similar total scores on Subtest 4; however, the PD-MCI group had the lowest total scores on Subtest 4 (Table 3).

**Table 3.** The performance of FER in each emotion category of the MMER app in study groups.

	HC (*n* = 63)	PD-NC (*n* = 21)	PD-MCI (*n* = 42)	Stat. value	*P* *	Post-hoc
***Subtest 3***						
Total (max. 30)	19.09 (4.49)	20.12 (4.23)	16.41 (5.01)	^a^11.359	.003	
Neutral (max. 4)	3.05 (1.10)	3.38 (0.74)	2.86 (1.03)	^a^3.810	.149	
Happy (max. 3)	2.92 (0.28)	2.90 (0.30)	2.76 (0.58)	^a^3.188	.203	
Sad (max. 3)	2.11 (0.63)	2.10 (0.77)	1.74 (0.66)	^a^7.805	.020	
Angry (max. 5)	4.27 (1.05)	4.19 (1.08)	3.69 (1.24)	^a^7.186	.028	
Disgust (max. 6)	4.35 (1.39)	4.52 (1.36)	3.57 (1.71)	^a^7.366	.025	
Fear (max. 6)	2.25 (1.28)	2.05 (1.40)	1.95 (1.43)	^a^3.188	.203	
Surprise (max. 3)	2.00 (0.86)	2.33 (0.73)	1.98 (0.90)	^a^2.645	.267	
**Subtest 4**						
Total (max. 20)	11.74 (3.26)	12.36 (2.85)	9.57 (3.32)	^b^7.647	.001	HC = PD-NC > PD-MCI
Neutral (max. 3)	1.76 (0.53)	1.76 (0.54)	1.71 (0.51)	^a^.659	.719	
Happy (max. 2)	1.94 (0.25)	1.95 (0.22)	1.90 (0.30)	^a^0.594	.743	
Sad (max. 3)	1.90 (0.86)	2.19 (0.81)	1.55 (1.02)	^a^6.940	.031	
Angry (max. 3)	2.54 (0.67)	2.52 (0.75)	2.19 (0.92)	^a^4.565	.102	
Disgust (max. 4)	3.03 (0.99)	2.95 (0.74)	2.02 (1.26)	^a^19.338	<.001	HC = PD-NC > PD-MCI
Fear (max. 3)	1.76 (1.30)	2.05 (1.07)	1.62 (1.06)	^a^0.594	.743	
Surprise (max. 2)	1.71 (0.52)	1.71 (0.46)	1.62 (0.62)	^a^0.511	0.774	

Notes: Please see Table 3 and MMER app, multi-modality emotion recognition mobile application for abbreviations.

a Kruskal–Wallis test;

b One-way ANOVA;

* Bonferroni correction was applied with a *P*-value <.0031.

### FER ability in each emotion category

Table 3 shows the performance of each group across the various emotion categories. We found a significant difference between the three study groups in the “disgust” score of Subtest 4 (*P* < .001). The post hoc revealed that the PD-NC and HC groups had similar scores, and the PD-MCI group had the lowest score in the “disgust” index (Fig. 2). We further analyzed the misclassification pattern in “disgust” FER performance in patients with PD in subtest 4. The results presented that the patients with PD-MCI were unable to accurately match the word “disgust” to the correct expression and often mismatched it to the negative emotion, such as “fear” (36%), “sad” (35%), and “angry” (4%).

Moreover, we present a full confusion matrix for all emotion categories in the MMER app (subtests 3 and 4) in Table 4 to offer a detailed view of emotion recognition patterns. This matrix shows how often each emotion was correctly identified or misclassified by the PD-MCI group, highlighting the specific challenges they faced. Notably, Table 4 reveals that “disgust” is frequently misidentified as “fear,” “sad,” or “angry,” underscoring the particular difficulty in recognizing “disgust” among PD patients with MCI.

**Table 4.** The reaction pattern of FER in each emotion category of the MMER app (subtest 3 + subtest4) in PD-MCI groups.

	Emotion categories						
Reaction pattern	Neutral (%)	Happy (%)	Sad (%)	Angry (%)	Disgust (%)	Fear (%)	Surprise (%)
Neutral	79.25	17.06	10.48	3.27	5.29	9.52	11.90
Happy	1.36	62.30	3.81	3.27	0.26	1.06	1.59
Sad	4.76	10.71	64.29	6.85	13.49	14.29	1.98
Angry	7.14	1.19	3.81	67.86	9.52	9.52	6.35
Disgust	3.40	1.98	9.05	10.71	62.17	6.88	3.97
Fear	2.04	1.19	4.29	2.98	6.88	41.01	8.33
Surprise	2.04	5.56	4.29	5.06	2.38	17.72	65.87

### Hierarchical regression of the prediction of cognitive function to predict the performance of FER in patients with PD

In Subtest 4, the total score can be predicted by “Attention” (i.e. Stroop-W), “Executive Function” (i.e. CTT-B), “Memory” (i.e. LM-I), and “Visual-Spatial” (i.e. JLO) (Table 5). This shows that the FER ability in Subtest 4 could be predicted from other cognitive domains besides “language.” In addition, the FER ability of “disgust” could be predicted by Stroop-C in “Attention,” CTT-B in “Executive Function,” LM-I in “Memory,” and JLO in “Visual-Spatial.”

**Table 5.** The hierarchical regression of the prediction of the cognitive functions to FER in the PD population.

		Attention/Working memory			Executive function				Language		Memory				Visual-Spatial	
MMER app		Stroop-w	Stroop-C	WAIS-DS	CTT-B	MCST-C	MCST-PE	MCST-NPE	VF	Language	LM-I	LM-II	VR-I	VR-II	JLO	pentagon
Subtest 3-Total	*R* ^2^	0.036	<0.001	0.003	0.031	0.002	0.034	0.009	<0.001	0.059	0.003	<0.001	0.046	0.004	0.044	0.020
	*F*	4.451	0.021	0.312	3.685	0.276	4.283	1.116	0.006	7.465	0.329	0.024	5.615	0.497	5.448	2.610
	*P*	.039*	.887	.579	.060	.601	.043*	.295	.937	.008**	.568	.877	.021*	.484	.023*	.112
Neutral	*R* ^2^	0.034	0.011	<0.001	0.008	0.001	0.009	0.003	0.003	0.089	0.007	0.058	0.060	<0.001	0.054	0.055
	*F*	2.500	0.829	0.023	0.600	0.069	0.627	0.224	0.208	6.968	0.509	4.372	4.857	0.003	4.095	4.390
	*P*	.119	.366	.881	.442	.794	.432	.637	.650	.011*	.479	.041*	.032*	.959	.047	.040*
Happy	*R* ^2^	0.005	0.022	<0.001	0.001	0.090	0.001	0.005	0.003	0.014	0.020	<0.001	0.005	0.014	0.019	0.001
	*F*	0.337	1.367	0.006	0.062	5.967	0.048	0.314	0.183	0.856	1.250	0.003	0.289	0.814	1.203	0.033
	*P*	.564	.247	.938	.804	.018*	.828	.578	.670	.358	.268	.959	.593	.371	.277	.857
Sad	*R* ^2^	<0.001	<0.001	<0.001	0.043	<0.001	0.002	<0.001	0.004	0.027	0.014	0.006	0.004	0.038	0.010	0.003
	*F*	0.012	0.007	0.012	3.146	0.011	0.136	0.005	0.275	1.928	0.989	0.408	0.247	2.677	0.688	0.225
	*P*	.914	.935	.913	.081	.916	.714	.944	.602	.170	.324	.525	.621	.107	.410	.637
Angry	*R* ^2^	0.026	0.005	0.005	0.002	0.003	0.043	0.005	0.002	0.077	<0.001	0.011	<0.001	0.004	0.027	0.002
	*F*	1.787	0.367	0.359	0.164	0.193	2.957	0.315	0.131	5.635	<0.001	0.754	0.013	0.230	1.856	0.109
	*P*	.186	.547	.551	.687	.662	.091	.577	.719	.021*	.993	.389	.910	.633	.178	.743
Disgust	*R* ^2^	0.078	<0.001	0.035	0.031	0.008	0.016	0.010	0.018	0.056	<0.001	0.001	0.064	<0.001	0.059	0.032
	*F*	6.665	0.479	3.088	2.444	0.648	1.274	0.794	1.390	4.654	0.024	0.106	5.211	0.007	4.891	2.771
	*P*	.012*	.492	.084	.123	.424	.264	.377	.243	.035*	.877	.746	.026*	.933	.031*	.101
Fear	*R* ^2^	0.015	<0.001	0.009	0.018	0.013	0.011	0.042	0.014	0.013	0.009	0.003	0.030	<0.001	0.020	0.013
	*F*	0.970	0.013	0.552	1.144	0.080	0.691	2.768	0.867	0.820	0.575	0.174	1.953	0.001	1.308	0.826
	*P*	.329	.909	.461	.289	.372	.409	.102	.355	.369	.451	.678	.168	.970	.257	.367
Surprise	*R* ^2^	0.026	<0.001	0.049	0.006	0.009	0.021	0.013	0.002	0.074	0.022	0.009	0.021	0.005	0.008	0.005
	*F*	1.684	0.005	3.318	0.400	0.596	1.340	0.858	0.100	5.056	1.440	0.577	1.389	0.291	0.508	0.319
	*P*	.199	.946	.074	.529	.443	.252	.358	.753	.028*	.235	.450	.243	.592	.479	.574
Subtest4-Total	*R* ^2^	0.051	0.017	0.004	0.088	0.005	0.014	0.001	<0.001	0.045	0.075	0.002	0.032	0.007	0.064	0.036
	*F*	4.969	1.666	0.384	9.115	0.482	1.437	0.074	<0.001	4.215	7.552	0.161	3.274	0.751	6.360	3.760
	*P*	.030*	.202	0538	.004**	.490	.236	.787	.994	.045	.008**	.690	.076	.390	.014*	.057
Neutral	*R* ^2^	0.019	<0.001	0.007	0.061	0.005	0.066	0.006	0.001	0.011	0.023	0.045	0.058	0.046	0.031	0.018
	*F*	1.280	0.013	0.435	4.185	0.316	4.744	0.427	0.044	0.723	1.487	3.046	4.130	3.467	2.048	1.224
	*P*	0.262	0.909	0.512	0.045	0.576	0.033*	0.516	0.834	0.399	0.227	0.086	0.047	0.068	0.158	0.273
Happy	*R* ^2^	0.007	0.012	0.015	0.017	0.040	0.004	<0.001	<0.001	<0.001	0.001	0.027	0.028	<0.001	<0.001	<0.001
	*F*	0.448	0.791	1.028	1.165	2.858	0.262	0.015	0.001	0.001	0.080	1.822	1.962	0.011	<0.001	0.001
	*P*	0.506	0.377	0.315	0.285	0.096	0.610	0.904	0.973	0.973	0.778	0.182	0.167	0.915	0.986	0.974
Sad	*R* ^2^	0.013	0.001	<0.001	0.006	<0.001	0.082	<0.001	0.025	0.024	0.012	0.010	0.026	<0.001	0.008	0.128
	*F*	0.841	0.080	<0.001	0.430	0.010	5.787	0.005	1.657	1.657	0.821	0.699	1.731	0.022	0.503	9.789
	*P*	.363	.779	.983	.514	.922	.019*	.942	.203	.203	.368	.406	.193	.883	.481	.003**
Angry	*R* ^2^	0.058	0.007	0.031	0.003	0.050	0.001	0.004	0.003	0.067	0.010	0.002	0.034	0.007	0.152	0.011
	*F*	3.909	0.454	2.139	0.191	3.316	0.048	0.263	0.174	4.518	0.614	0.123	2.210	0.433	11.457	0.827
	*P*	.053	.503	.149	.663	.074	.828	.610	.678	.038*	.436	.728	.143	.513	<.001***	.367
Disgust	*R* ^2^	0.055	0.093	0.001	0.159	0.007	0.001	0.012	0.049	0.029	0.145	0.003	0.001	0.048	0.112	0.001
	*F*	4.012	7.472	0.077	13.289	0.575	0.076	1.016	3.534	2.122	11.862	0.236	0.119	4.045	8.720	0.056
	*P*	.050	.008**	.782	<.001***	.451	.784	.318	.065	.151	<.001***	.629	.732	.049	.005**	.813
Fear	*R* ^2^	0.036	0.012	0.003	0.001	0.012	0.002	0.008	0.002	0.007	0.001	<0.001	0.011	0.006	0.003	0.067
	*F*	2.255	0.747	0.170	0.035	0.745	0.109	0.459	0.124	0.422	0.067	<0.001	0.635	0.380	0.213	4.310
	*P*	.138	.391	.682	.853	.391	.743	.501	.726	.518	.796	.990	.429	.540	.646	.042*
Surprise	*R* ^2^	0.004	0.001	0.006	0.044	0.009	0.041	0.032	<0.001	0.011	0.093	0.024	0.046	0.002	0.027	0.001
	*F*	0.287	0.044	0.381	3.034	0.647	2.896	2.327	0.025	0.715	6.838	1.767	3.592	0.169	1.814	0.050
	*P*	.594	.835	.539	.087	.424	.094	.133	.874	.401	.011*	.189	.063	.682	.183	.823

Notes: Please see Tables 2–4 for abbreviatons.

* *P* < .05,

** *P* < .01,

*** *P* < .001.

### Mediation model

**Figure 3.**
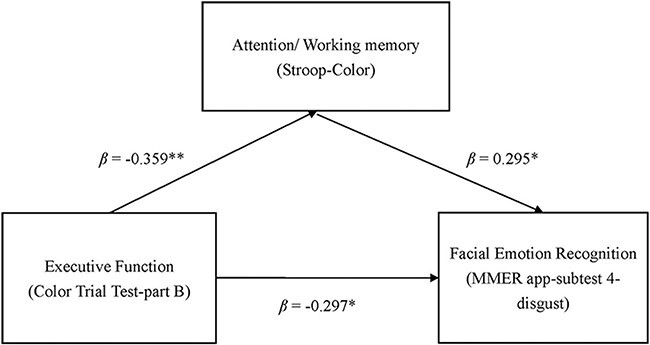
The mediation model of the executive function, attention, and FER.

After knowing that most domain indicators of cognitive function predicted “disgust” FER performance in subtest 4, we proceeded with a mediation analysis (Table 6 and Fig. 3).

1. After controlling the age and sex, the executive function index (CTT-B) can predict the performance of MMER app-subtest 4-disgust (β = −0.403, *P *= .001; Model 4).
2. After controlling for age and sex, the executive function index (CTT-B) can predict the performance of the attention index (β = −0.359, *P *= .002; Model 2).
3. After controlling for age and sex, the attention index (Stroop-C) can predict the performance of MMER app-subtest 4-disgust (β = 0.597, *P *= .001; Model 5).
4. After controlling for age and sex, we also examined the potential mediating effect of the attention index (Stroop-color) on the relationship between the executive function index (CTT-B) and performance in the MMER app-subtest 4-disgust. The analysis revealed that the attention index partially mediated this relationship (β = −0.297, *P* = .013; Model 6).

**Table 6.** The regression model of executive function, attention/ working memory, and the MMER app-subtest 4-disgust.

	Mediator		Dependent variable			
	Attention/Working memory		FER (MMER app-subtest 4-disgust)			
	Model 1	Model 2	Model 3	Model 4	Model 5	Model 6
Control variable						
Sex	0.047	0.061	−0.233	−0.217	−0.253*	−0.235*
Age	−0.431**	−0.387**	−0.248	−0.198	−0.066	−0.084
Independent variable						
Executive function (CTT-B)		−0.359**		−0.403**		−0.297*
Mediator						
Attention/Working memory (Stroop-C)					0.597**	0.295*
*R* ^2^	0.182	0.309	0.133	0.292	0.279	0.352
Adj. *R*^2^	0.155	0.274	0.104	0.256	0.243	0.308
*△R* ^2^	0.182	0.127	0.133	0.159	0.147	0.220
*F*	6.693**	8.803***	4.597*	8.122***	7.626***	7.894***
*Df*	(2, 60)	(3, 59)	(2, 60)	(3, 59)	(3, 59)	(4, 58)

Notes: The values in the table are standardized *β*. df, degree of freedom;

* *P* < .05,

** *P* < .01,

*** *P* < .001.

## Discussion

The main findings of this study were as follows: (i) the FER performance of the PD-NC group was comparable to that of healthy controls. (ii) The PD-MCI group had poorer FER performance than the PD-NC and HC groups, particularly in recognizing “disgust” emotions. (iii) Cognitive function indices, including attention, executive function, memory, and visual-spatial function, can predict FER in patients with PD. (iv) The impact of executive function on disgust FER is partially mediated by attention.

### Patients with PD’s general and emotion-specific FER

We found that the FER is impaired in PD-MCI, and this deficit was found in recognizing disgust on the faces of others. Our findings were consistent with prior research (Pietschnig et al. 2016, Waldthaler et al. 2019, Galtier et al. 2021).

Our study revealed a significant deficit in recognizing disgust facial emotions in individuals with PD-MCI, which aligns with previous research (Suzuki et al. 2006, Argaud et al. 2018), suggesting that FER defects in PD are specific to certain emotion categories. However, Alonso-Recio et al. (2014) and Waldthaler et al. (2019) found no significant difference in specific emotion recognition among patients with PD-MCI, PD-NC, and healthy controls (Waldthaler et al. 2019); However, the Waldthaler et al.’s (2019) small sample size (*n* = 12) limited its external validity. Neurophysiological evidence suggests that dopamine neuron deficits in PD are initially limited to the ventral substantia nigra, which mainly affects the caudate nucleus and limbic region involved in emotional function (Péron et al. 2012). Previous research has identified the cingulate gyrus in the limbic system as a crucial area for “disgust” FER (Ibarretxe‐Bilbao et al. 2009) indicating deficits in recognizing disgust faces may manifest earlier in PD than deficits in recognizing other emotions. Wicker et al. (2003) found that “disgust” involved the left anterior insula and right anterior cingulate cortex (Wicker et al. 2003). Among them, the front to the middle part of the insula is the most significant (Motomura et al. 2019), and the anterior insula participates in the cingulo-opercular network (Dosenbach et al. 2008).

Additionally, we did not observe any significant differences in “disgust” FER in Subtest 3. One potential explanation for this finding is that the contents of the two subtests were dissimilar. Under typical circumstances, individuals respond quickly to social cues. Subtest 4 was more closely aligned with real-world scenarios than Subtest 3, thus displaying greater ecological validity. Therefore, we contend that Subtest 4 is more capable of detecting deficits in disgust FER among individuals with PD.

### Relationship between cognitive function and FER performance in patients with PD

Previous studies have suggested that patients with PD’s general FER may associated with executive function (Mattavelli et al. 2021), visual-spatial function (Ho et al. 2020), and attention (Dujardin et al. 2013, Alonso-Recio et al. 2014, Wallace et al. 2022, Burgio et al. 2024). Our findings align with those of previous studies and suggest that FER is related to attention, executive function, memory, and visuospatial function, and that these cognitive functions can predict general FER ability in patients with PD. Our results also indicated that FER performance in the negative emotion category correlated with all cognitive function domains, which is consistent with previous research (Ho et al. 2020, Mattavelli et al. 2021).

Notably, recognizing “disgust” emotions in patients with PD relates to multiple cognitive functions; attention, executive function, memory, and visual-spatial function can all predict “disgust” FER ability. The anterior insula, which receives and processes perceptions from other cortexes, is a critical brain region affecting “disgust” FER performance, and it is linked with other brain regions involved in sensory, motor, and autonomic functions, as well as various cognitive functions (Chang et al. 2013, Motomura et al. 2019). The similarities in facial muscle movements between different emotion categories, such as “anger” and “disgust,” can also make it difficult to recognize “disgust.” For example, both “anger” and “disgust” involve raising the eyelids and wrinkling the nose, as observed in previous studies (Poncet et al. 2021).

### Attention partially mediated the relationship between executive function and FER

We found that executive function in patients with PD significantly predicted FER performance, specifically in recognizing disgust. Additionally, attentional factors affected FER performance. Visual attention, which is related to the spatial organization of the head and eye exploration, also influenced FER. The frontal lobe controls visual attention, head spatial organization, eye exploration, and executive function (Circelli et al. 2013). Circelli et al. (2013) supported this finding, indicating that individuals with poorer executive function may spend more time scanning and analyzing negative emotional faces to recognize them accurately(Circelli et al. 2013).

Attention is a crucial cognitive function that significantly affects recognition. In patients with PD, dopamine depletion in the limbic circuits often links the striatum to the frontal and prefrontal regions, which is believed to be the primary reason for attention and executive function dysfunction. Furthermore, Dujardin et al. (2013) highlighted that patients with PD have deficits in their supervisory attentional system, making it challenging and time-consuming to use cognitive flexibility to resolve conflicts between multiple stimuli. Consequently, PD individuals may experience difficulty accurately recognizing emotions (Dujardin et al. 2013).

This study revealed that cognitive flexibility (i.e. CTT-B) was partially mediated by focused attention (i.e. Stroop-Color) when predicting the recognition of “disgust” facial expressions in patients with PD. Our mediated model indicated that cognitive flexibility initially affected attention and subsequently influenced the recognition of “disgust” facial expressions in patients with PD. Cognitive flexibility involves the ability to switch between concepts and cues. Patients with PD and poor cognitive flexibility may struggle to shift and maintain their attention on the necessary facial cues (e.g. frown or wrinkled nose), leading to impaired recognition of “disgust” emotions. Patients with PD and relatively good cognitive flexibility may still have difficulties reorganizing “disgust” facial emotions.

Improving the FER in patients with PD can be facilitated by training in cognitive flexibility and attentional focus. Recognizing the “disgust” facial emotion requires a focus on the lower portion of the face, particularly the mouth area (Beaudry et al. 2014). However, other facial features, such as a frown, wrinkled nose, lifted chin, and parted lips of the face, are also critical elements in recognizing “disgust” emotion (Kuramoto et al. 2019, Poncet et al. 2021). Patients with PD need to use cognitive flexibility to shift their attention from the mouth to other critical areas of the face and focus on cues to collect and analyze information from different facial cues successfully.

### Constraints on generality

The study included participants with PD who received treatment at two medical centers in southern Taiwan. All participants were Ethnic Chinese and lived in densely populated areas, providing them with ample opportunities to interact with others. The FER stimuli used in this study were faces of Ethnic Chinese people, and emotional words were presented in traditional Chinese characters. These findings may affect the highly industrialized, urbanized, and Ethnic Chinese populations.

### Limitations and future studies

This study has several limitations that need to be acknowledged. First, a causal relationship between cognitive function and FER could not be established owing to the cross-sectional design. Second, the lack of ecological validity may limit the generalizability of our findings. Although this investigation employed a robust and rigorous emotion recognition assessment, it is crucial to acknowledge that the static facial images utilized in the study deviated from the dynamic and nuanced nature of emotional stimuli encountered in everyday real-world scenarios. Thirdly, we found that some PD-NCs may have lower FER, which raises the question of investigating FER in the “pre PD-MCI” stage to early detection. Lastly, although we found an effect of cognitive function on FER in patients with PD, the specificity of the impairment in recognizing “disgust” emotions in PD-MCI needs to be compared with that of MCI patients without PD to distinguish the effects of PD and cognitive impairment; however, participant’s recruitment during the COVID-19 pandemic has been difficult. Further research is required to provide a more comprehensive understanding of the pathophysiological aspects of impaired FER in patients with PD-MCI.

## Conclusions

The FER ability of patients with PD-NC was similar to that of healthy controls matched for age, sex, and education. However, patients with PD-MCI had impaired FER, particularly in recognizing “disgust” facial emotions. Furthermore, attention was found to partially mediate the relationship between executive function and recognition of “disgust” facial emotions. We propose that adjusting attention could influence the effect of executive function on the recognition of “disgust” emotions in facial expressions. These findings provide a foundation for cognitive training to improve FER in the future.

## Data Availability

We report how we determined our sample size, all data exclusions (if any), all manipulations, and all measures in the study, and we follow JARS (Kazak 2018). Data were analyzed using the 26th edition of Statistical Product and Service Solutions statistical software. This study’s Materials and analysis codes are unavailable due to ethical problems. The study’s design and its analysis were not preregistered.
